# Proton MR spectroscopy of human pancreas allografts

**DOI:** 10.1007/s10334-019-00740-8

**Published:** 2019-04-01

**Authors:** Jan Weis, Håkan Ahlström, Olle Korsgren

**Affiliations:** 1 Department of Medical Physics, Uppsala University Hospital, 751 85 Uppsala, Sweden; 2 Department of Radiology, Uppsala University Hospital, Uppsala, Sweden; 3 Rudbeck Laboratory, Department of Immunology, Genetics and Pathology, Uppsala University Hospital, Uppsala, Sweden

**Keywords:** ^1^H-MRS, Pancreas graft, Relaxation times, Total fat, Intracellular fat

## Abstract

**Objective:**

To estimate pancreas graft relaxation times and concentrations of total fat, and the intracellular lipids of non-adipose pancreatic cells (NAPC) using proton (^1^H) magnetic resonance spectroscopy (MRS) during cold preservation.

**Materials and methods:**

Grafts from 11 human donors were investigated. Each pancreas was perfused in situ with histidine-tryptophan-ketoglutarate (HTK) or with University of Wisconsin solution and placed into a transport container. Temperature of the grafts was maintained at 4 ± 2 °C during transport to our hospital and MR scanning. A 1.5 T clinical scanner was used for the measurements. Single-voxel PRESS spectra were acquired using transmit–receiver head coil.

**Results:**

Relaxation times were measured for lipid (–CH_2_–)_n_ (*T*_1_, 287 ± 60 ms; *T*_2_, 27 ± 4 ms), and tissue water (*T*_1_, 670 ± 69 ms; *T*_2_, 77 ± 17 ms). Average total fat, and intracellular lipids of NAPC concentrations were 79.2 ± 100.8 (range 2.4–304.4), and 2.9 ± 1.2 mmol/kg ww, respectively.

**Conclusion:**

We have shown that ^1^H-MRS is a useful tool for the estimation of pancreas graft lipid concentrations. Total pancreatic fat and especially content of intracellular lipids of NAPC are valuable measures for inspection of graft quality prior to transplantation or islet of Langerhans isolation.

## Introduction

The pancreas plays an important role in the synthesis and secretion of digestive enzymes and metabolism-regulating hormones. The majority of pancreas volume contains exocrine glands that are dedicated to producing enzymes which help to digest proteins, fats, and carbohydrates (sugars). Approximately 2% of the pancreas mass consists of different hormone-producing endocrine cells which clump together into small clusters called islets of Langerhans. The hormones made by alpha and beta cells in the islets produce glucagon and insulin, respectively. These two hormones regulate the sugar levels in the blood and cells.

Pancreas transplantation is the treatment of choice for patients whose pancreas does not make enough, or sometimes any, insulin. These patients suffer type 1 diabetes, or insulin-dependent type 2 diabetes. A relatively new and minimally invasive therapy is transplantation of the islets of Langerhans [1, 2]. Islets are delivered into the liver by injection to the portal vein, where they produce insulin. Pancreas or islets transplantation is currently the only treatment that restores normal glucose metabolism in insulin-dependent patients [3, 4].

The pancreas is regarded as one of the most challenging donor organs for recovery and transplantation. The selection of deceased donors for pancreas procurement is the key factor of outcome in transplantation of the whole organ or islets. The ideal donor developed brain death as a result of trauma, ranges in age from 10 to 40 years and has a body mass index (BMI) less than 27.5 kg/m^2^ [5]. Such strict donor selection has not only resulted in outstanding graft survival but also in underutilization of pancreas donors [6]. Fortunately, recent successes in pancreas transplantation have led to the utilization of less-than-ideal donors. It was demonstrated that graft survival was not significantly different in patients receiving transplants from obese, non-heart beating or younger donors compared with grafts from ideal donors [5, 6]. However, the use of less-than-ideal donors requires methods for an objective assessment of pancreas graft quality. The aim of such methods is to predict either pancreas donor utilization or graft failure. Possible methods of choice are phosphorus (^31^P) [7, 8] and proton magnetic resonance spectroscopy (^1^H-MRS) [9–12].

^1^H-MRS is a promising tool for non-invasive quantification of pancreatic fat content. Fat assessment is an important issue because fatty pancreas is more difficult to procure, and prepare on backbench. In addition, excessive fatty infiltration is associated with an increased risk of complications after pancreatic surgery [5]. BMI seems to be a less reliable measure of pancreas fat content [9, 13]. Some overweight donors may have little or no fatty infiltration of the pancreas, while some donors with normal BMI may have increased fat content [5, 10, 14]. Excessive alcohol abuse may also increase pancreatic lipid content, even in the absence of obesity [5, 10].

Relatively few in vivo ^1^H-MRS studies of human pancreas have been performed [9–12] so far. Lipids and total choline (tCho, free choline, phosphocholine, and glycerophosphocholine) were quantifiable despite experimental difficulties with respiratory motion. It was shown that pancreatic fat content was increased in men with type 2 diabetes [9, 10, 15]. In non-diabetic men the fat content was inversely associated with various indicators for β-cells functions [9, 14]. It should be noted that ^1^H-MRS is able to distinguish between lipids in fat cells (adipocytes) and lipids in non-adipose cells (triglyceride droplets in the cytosol). Examples are intramyocellular [16], intrahepatic [17], intramyocardial lipids [18], and intracellular lipids of non-adipose pancreatic cells (NAPC).

In the present study, we applied ^1^H-MRS to human pancreas grafts during cold preservation. First, we measured pancreatic water and fat relaxation times *T*_1_ and *T*_2_. Second, knowledge of the relaxation times enabled us to estimate the absolute total fat concentration and intracellular lipids of NAPC. Third, we evaluated the relationship between pancreatic total fat content and donor’s BMI.

## Materials and methods

### Donors

Pancreas grafts from 11 human donors were included in this study. Donor’s characteristics are shown in Table 1. Organ donation was performed solely for research purposes and was approved by the relatives. We note that the grafts of donors were considered unsuitable for organ transplantation due to the advanced age (nine grafts) or for other reasons (two grafts). The study was approved by the Regional Ethical Review Board in Uppsala. Ten pancreas grafts were perfused in situ with histidine-tryptophan-ketoglutarate (HTK) solution and one graft was perfused with University of Wisconsin (UW) solution. After the perfusion, the pancreata was stored in a plastic container (MEDCO AS, Årvollskogen, Norway) filled with HTK or UW solution. The temperature inside the container was maintained at 4 ± 2 °C during the transport to our hospital and MR scanning.

**Table 1:** Gender, age, BMI of the donors and lipid concentrations of pancreas grafts

Donor nr	Gender	Age (years)	BMI (kg/m^2^)	Concentration			
				Total lipid content		Intracellular lipids of NAPC	
				f/w (%)	mmol/kg ww	f/w (%)	mmol/kg ww
1	M	56	29.2	26.61	304.38	–	–
2	F	62	29.4	0.83	9.53	0.36	4.07
3	M	24	31.9	0.21	2.4	0.12	1.36
4	M	68	24	0.89	10.17	0.32	3.68
5	F	63	30.9	2.78	31.8	–	–
6	M	28	24.5	7.77	88.93	–	–
7	M	79	22.2	5.49	62.77	–	–
8	F	70	23	0.38	4.34	0.21	2.38
9	F	82	31.3	4.71	53.85	–	–
10	M	70	23	21.06	240.97	–	–
11	M	50	22.3	5.48	62.55	–	–
Mean		59.3	26.5	6.93	79.24	0.25	2.87

*NAPC* non-adipose pancreatic cells, *f/w* fat/water spectral intensity ratio

### Data acquisition

**Fig. 1:**
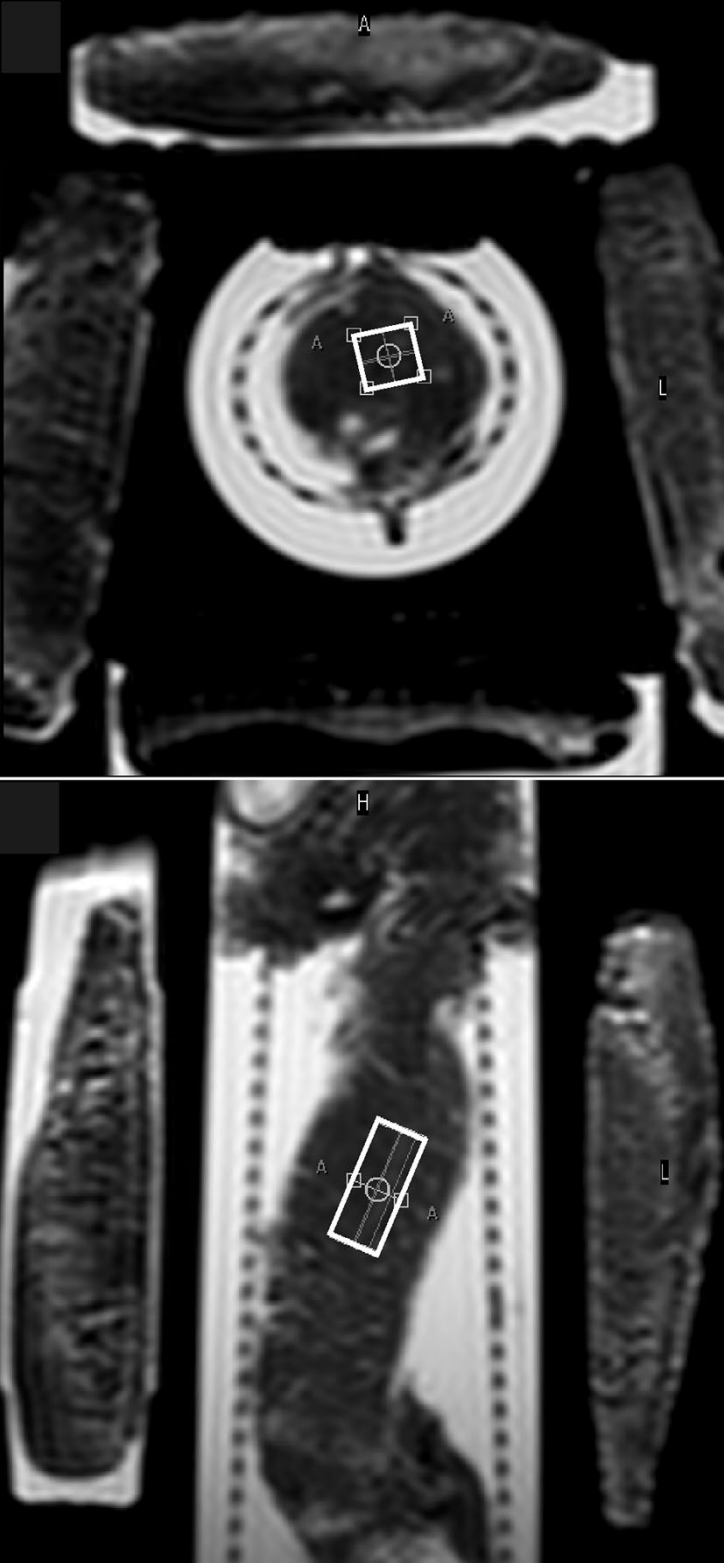
*T*_2_-weighted MR images showing typical voxel position in the body of the pancreas. Cylindrical transport container was surrounded by four cooling (ice) elements

Experiments were performed on a Philips Achieva 1.5 T MR scanner (Philips Healthcare, Best, The Netherlands). ^1^H-MRS was performed using the standard transmit–receiver quadrature head coil. *T*_2_-weighted images were applied for voxel positioning. The voxel was placed within the body of the pancreas (Fig. 1). Voxel size was between 10 × 10 × 15 and 10 × 10 × 25 mm^3^. Proton spectra of HTK and UW solutions as well as spectra determined for lipid quantitation were acquired by point-resolved spectroscopy (PRESS) sequence (time repetition TR 5000 ms, time echo TE 30 ms, spectral bandwidth 1000 Hz, 1024 points, 16 phase cycle steps). Two dummy excitations were followed by 16 non-water-suppressed and 64 water-suppressed scans. Net acquisition time was 6 min and 50 s.

Water relaxation times *T*_1_ and *T*_2_ were computed using the PRESS spectra acquired at ten TRs (300, 350, 400, 500, 750, 1000, 1500, 2000, 3000, 5000 ms, number of scans (NS) 16, TE 30 ms), and eight TEs (30, 40, 50, 75, 100, 125, 150, 200 ms, TR 3000 ms, NS 16). The *T*_1_ values of lipid (–CH_2_–)_n_ line was quantified from the spectra measured at TE 25 ms and TRs 550, 650, 750, 850, 1000, 1200, 1400, and 1600 ms (NS 64 or 128). The *T*_2_ value was estimated using the spectra measured at TEs 30, 40, 50, 60, 80, 100, 120, and 150 ms (TR 1500 ms, NS 64 or 128).

The pancreas was measured in its original cylindrical transport container. During scanning hypothermic storage was maintained by three or four cooling elements (Fig. 1). Temperature stability (4 ± 2 °C) was checked in five grafts by measuring the temperature of the HTK or UW solution inside the transport container just before and immediately after MR examination.

### Spectrum processing

**Fig. 2:**
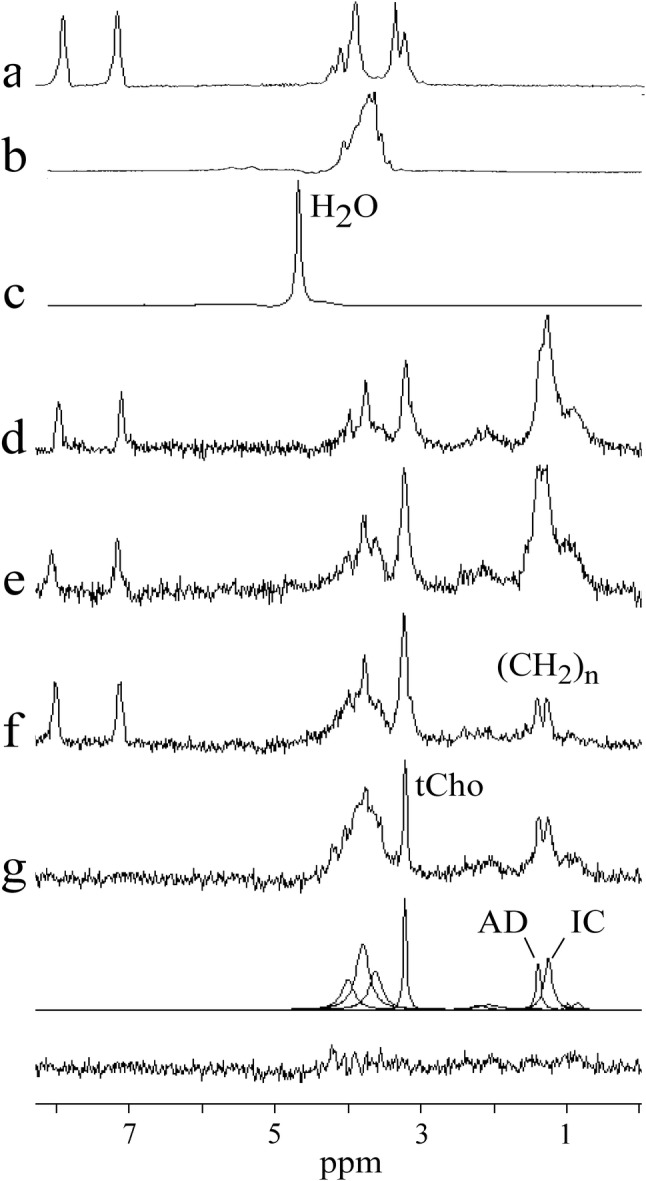
**a**^1^H spectrum of HTK solution (histidine). **b** Spectrum of UW solution (raffinose). **c–f** Spectra of pancreas grafts perfused with HTK solution. **c** Unsuppressed water line (concentration reference) of spectrum **d**. **g** Spectrum of pancreas graft perfused with UW solution, fits and residue. Resonance at 1.3 ppm represents intracellular (IC) lipid methylene line of NAPC. Methylene peak at 1.42 ppm originates from pancreatic adipocytes (AD)

^1^H spectra were fitted in time domain using AMARES algorithm [19] as implemented in the magnetic resonance user interface (MRUI) software package [20]. After manual phase correction, the residual water line was removed with Hankel–Lanczos singular values decomposition (HLSVD) filter. Spectral lines were fitted with AMARES to Lorentzian line shapes. No apodization of free induction decay was used in this study. Prior knowledge applied for fitting the lipid signals originated from adipocytes and intracellular lipids of NAPC has been described elsewhere [21]. Spectral line at 3.2 ppm (tCho and histidine lines of HTK solution) was fitted by single Lorentzian. Spectral intensities between 3.5 and 4.2 ppm were empirically fitted by three Lorentzians (Fig. 2).

**Fig. 3:**
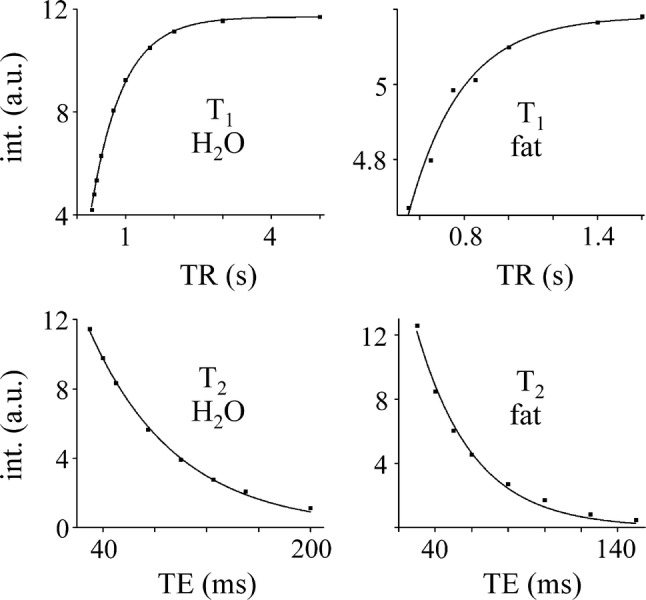
Monoexponential fits of spectral intensities vs. TR and TE

Relaxation times *T*_1_ and *T*_2_ were estimated by monoexponential fitting of the spectral intensities (Fig. 3) by a Levenberg–Marquadt algorithm using the commercial software package ORIGIN v. 8.6 (OriginLab, Northampton, MA, USA). Spectral intensities versus TE or TR were fitted using the PRESS relaxation attenuation function att(*T*_1_, *T*_2_) = exp(− TE/*T*_2_) × [1 − exp(− TR/*T*_1_)].

Lipid content was computed from the relaxation-corrected spectral intensity ratios to unsuppressed water line [22, 23]. Reference concentration 38 300 mM of “NMR-visible” water was used in the estimation of the absolute concentration. The value 38 300 mM was computed using the assumption that pancreas contains 0.71 g H_2_O per 1 g wet weight tissue [12] and its density is 1.08 g/cm^3^ [24]. Furthermore, it was assumed that 10% of tissue water is “NMR-invisible” because of macromolecular binding and other interactions [25]. Lipid content was quantified using methylene intensities. Division by factor 31 was used to convert methylene concentration to molecular concentration. The value 31 follows from the assumption that the average number of (–CH_2_–)_n_ groups is 31 per fat (triglyceride) molecule [23]. Division by the pancreas density was used to convert mM to millimoles per kg wet weight (mmol/kg ww). Total lipid content was also expressed as the ratio of lipid (–CH_2_–)_n_ over water spectral intensity (f/w) for completeness and in line with the previous studies [9, 10].

### Statistics

Quantitative results are presented as means ± 1 SD. Adjusted coefficient of determination adj. *R*^2^ was used to express goodness of nonlinear fits. Linear regression was performed to evaluate the relationship between BMI and pancreatic total fat content.

## Results

The pancreas grafts were delivered to our hospital between 5.5 and 12.5 h (median 8 h) after the start of perfusion with HTK or UW solution. All the spectroscopic experiments were performed within a few hours after delivery. Figure 2a, b shows the spectra of HTK and UW solutions, respectively. Only the spectral lines of the HTK’s histidine (180 mM) and the UW’s raffinose (30 mM) are detectable. Figure 2c depicts the unsuppressed water line (concentration reference) of spectrum shown in Fig. 2d. Methylene (–CH2–)_n_ spectral lines of intracellular lipids were quantifiable in four spectra (Fig. 2d–g) with low total lipid content (f/w ≤ 0.9%). The intracellular methylene resonance was unresolvable from methylene peak of pancreatic adipocytes in spectra with increased fat content (not shown). Figure 2d–f show the pancreas spectra perfused by HTK solution. The spectrum of pancreas perfused by UW solution is shown in Fig. 2g. This spectrum is completed by fits and residue. Resonance at 1.3 ppm represents intracellular (IC) lipid methylene line of NAPC. Methylene peak at 1.42 ppm originates from pancreatic adipocytes (AD). From Fig. 2d–f follows that tCho (3.2 ppm) is overlapped by strong signals of histidine doublet of doublets. In contrast, tCho is clearly separated from neighboring resonances in Fig. 2g. The intensities between 3.4 and 4.2 ppm were fitted by three Lorentzians. These fits refer to an empirical attempt to compensate for the signals in this region containing a large number of spectral lines with a high degree of overlap. Histidine (HTK solution) and raffinose (UW solution) dominate in this spectral interval. However, glucose, glycerol, choline, phosphocholine, glycerophosphocholine, myo-Inositol, ethanolamine, uridine, and amino acids were also identified in this region using high-resolution magic angle spinning (HR-MAS) spectroscopy [26].

**Fig. 4:**
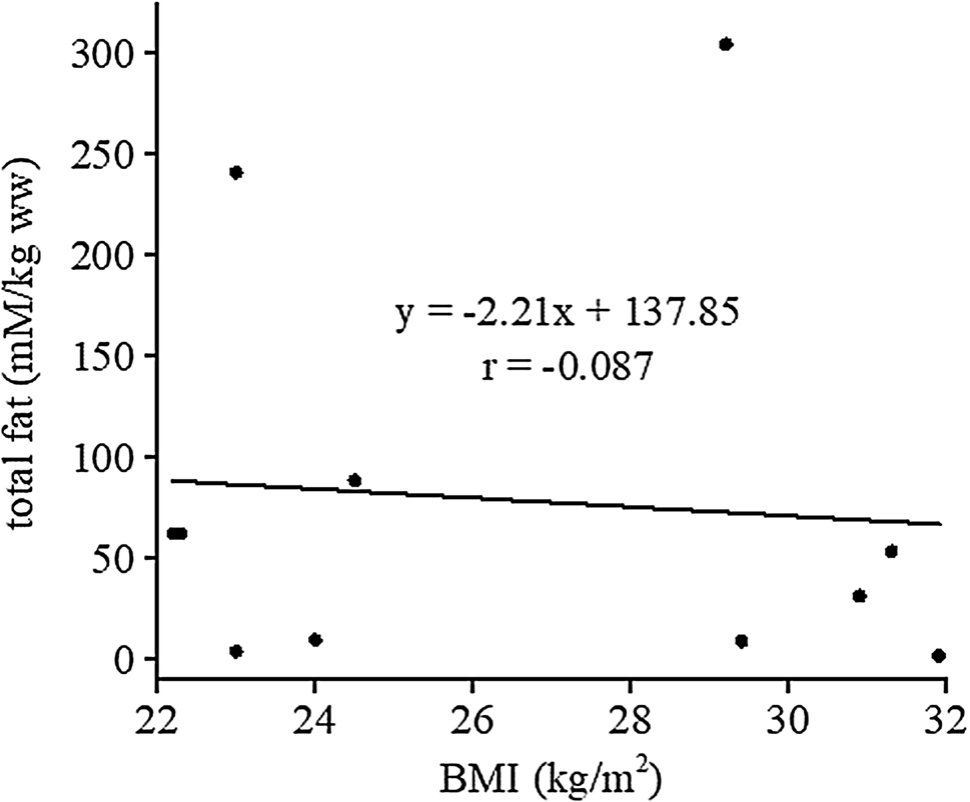
Scatter plot and regression line illustrating relationship between BMI and total pancreatic lipid content

Excellent match of spectral intensities to monoexponential functions was achieved for water relaxation times *T*_1_, *T*_2_ (adjusted *R*^2^ ≥ 0.991). Adjusted *R*^2^ between 0.846 and 0.984 was achieved for fat *T*_1_. Spin–spin relaxation time *T*_2_ of the lipid methylene spectra line was fitted within the interval 0.985 ≤ adj. *R*^2^ ≤ 0.992. Relaxation times are summarized in Table 2. Concentrations of total fat and intracellular lipids of NAPC are shown in Table 1. No correlation (*r* = − 0.087) was found between total fat content and BMI (Fig. 4).

**Table 2:** Average *T*_1_ and *T*_2_ relaxation times (ms) of normal pancreas grafts at 4 ± 2 °C

	Water (*N* = 5)	Fat (CH_2_)_n_ (*N* = 5)
*T*_1_	670 ± 69	287 ± 60
*T*_2_	77 ± 17	27 ± 4

*N* number of grafts

## Discussion

To our knowledge, this is the first ^1^H-MRS study of human pancreas grafts. For the first time relaxation times were measured during the cold preservation (~ 4 °C) and for the first time intracellular lipids of non-adipose pancreatic cells were detected and quantified by ^1^H-MRS.

In vivo quantitation of intracellular lipids of NAPC is a difficult task. Navigator-guided acquisition, respiratory triggering, or measurement during breath-hold are able to suppress spectral distortions, however, remaining residual respiratory, peristaltic and heart motion could cause additional phase and frequency distortions and anomalies in water suppression [27]. Spectroscopy of the pancreas grafts during cold storage offer the unique opportunity to obtain spectra free from motion artifacts. However, the absolute quantitation of intracellular lipids of NAPC is not always possible in spite of motion artifact-free spectra. Quantification depends on the ability of fitting algorithm to distinguish methylene spectral line of intracellular lipids at 1.3 ppm from the methylene line of adipocytes at ~ 1.4 ppm. Already modest increase of pancreatic adipocytes produces dominant lipid (–CH_2_–)_n_ signal which contaminate the intracellular spectral line due to severe overlap. Intracellular lipids are then indistinguishable. Increased spectral resolution at higher magnetic fields (*B*_0_ ≥ 7 T) can substantially improve detectability of intracellular lipids. Total Cho peak at 3.2 ppm is also undetectable when the lipid peak is high.

In vivo estimation of pancreatic water relaxation times *T*_1_ (584 ± 14 ms and *T*_2_ (46 ± 6 ms) was published by de Bazelaire et al. [28]. Our shorter *T*_1_ and longer *T*_2_ values can be explained by the fact that the pancreatic blood was replaced by HTK or UW solution. Measured *T*_1_ relaxation time of lipid (–CH_2_–)_n_ peak is shorter, about ~ 70 ms, and *T*_2_ is by factor ~ 2 smaller compared to the reported in vivo values of subcutaneous fat [28, 29]. This phenomenon can be explained by the low temperature during the experiments (4 ± 2 °C). Baron et al. [30] reported that *T*_1_ temperature (*T*) dependence of female breast adipose tissue can be described by the function *A *× exp[− *B*/(*T* + 273.15)] where *A* and *B* are constants. *T*_1_ temperature coefficient decreased from d*T*_1_/d*T* = 9.5 ± 0.16 ms/°C to 5.35 ± 0.08 ms/°C in the interval from 65 to 25 °C. Linear dependence (0.9 ± 0.03 ms/°C) was found between *T*_2_ and temperature. These *T*_1_, *T*_2_ dependences and the temperature difference ~ 33 °C between in vivo and cold storage explain the shorter relaxation times.

In this study, we found the pancreatic fat content (f/w) to be very similar, to those reported for healthy subjects [9, 10, 12, 13]. Spectroscopic voxel was placed within the body of the pancreas (Fig. 1) because of the need for large voxel size and because no significant [31] or small [15] difference of the fat content was reported between the head, body, and tail. No correlation was found between fat content and BMI. This result is in agreement with the previous studies of Tushuizen and Gaborit et al. [9, 13]. However, Lingvay et al. [10] and Kühn et al. [15] found a positive association of pancreatic fat content with BMI.

Pancreatic intracellular lipid accumulation is currently of considerable interest. It was shown that an increased content of cytosolic lipid droplets in islets of Langerhans leads to decreased glucose-stimulated insulin secretion [32, 33]. This phenomenon has been called as β-cell lipotoxicity [34]. To our knowledge, there are no previous estimates of human pancreatic intracellular lipids of NAPC that can be compared with our results. The intracellular lipid content we found is comparable with intramyocellular lipids of the calf muscles [21, 35, 36]. It is important to note that ^1^H-MRS is unable to discriminate between intracellular lipids of acinar and the islet of Langerhans cells.

The main limitation of this study is the small number of grafts and age of donors. It should be noted that the grafts were considered unsuitable for transplantation mainly due to advanced age. A further limitation is the fact that increased pancreatic fat content hinders detection of intracellular lipids of NAPC. The usability of the measured relaxation times is limited to the grafts perfused by HTK or UW solution and to the temperature of cold storage (4 ± 2 °C).

## Conclusions

The present results suggest that ^1^H-MRS is a useful tool for quantification of pancreas graft lipid concentrations using water as the internal concentration reference. Total pancreatic fat and especially content of intracellular lipids of NAPC are valuable measures for inspection of graft quality prior to transplantation or islet of Langerhans isolation.
